# Metastatic Gas gangrene and Colonic Perforation: a case report

**DOI:** 10.1186/1749-7922-3-15

**Published:** 2008-03-28

**Authors:** Matthew J Powell, Kishore K Sasapu, Christopher Macklin

**Affiliations:** 1 Mid Yorkshire NHS trust, Pinderfields General Hospital, Wakefield, Yorkshire, UK

## Abstract

*Clostridium septicum *myonecrosis is associated with diabetes, colorectal and haematological malignancies. We present a case of metastatic myonecrosis in a diabetic patient with a perforated caecal tumour. The literature since 1989 is reviewed and 28 cases of *Clostridium septicum *myonecrosis are discussed.

## Background

*Clostridium *infection presents as either septicaemia or myonecrosis, and can be classified as traumatic or non-traumatic. *Clostridium *species are sporulating Gram-positive rods identified by the Nagler reaction, which detects α toxin, a phospholipase [1]. The commonest organisms causing bacteraemia are *Clostridium perfringens *and *Clostridium septicum*. *Clostridium septicum *is found in soil and animal intestines, and is an opportunistic pathogen in humans, unlike *Clostridium perfringens*, which is part of the normal bowel flora. Infection is characterised by intense pain, a brown discolouration of the skin with violaceous bullae formation, gas in the soft tissues, an odourless discharge and necrosis of the muscles. The patients are unwell and have a low grade fever and tachycardia. Hyperkalaemia and renal failure often prove fatal before diagnosis is made. *Clostridium septicum *infection occurs most commonly in diabetics, the immunosuppressed, and is associated with intra abdominal and haematological malignancies [2-4].

## Case presentation

A fifty eight year old woman, who had well controlled type II diabetes, had been seen in the gynaecology outpatients department with a pelvic mass of unknown origin. She had refused transvaginal examination or ultrasound. She was admitted acutely by her General Practitioner with a short history of increasing abdominal pain.

On admission she was noted to be anaemic and had mildly raised inflammatory markers, white cell count 12 and C – reactive protein 20. Whilst awaiting further investigation her clinical condition rapidly deteriorated. She developed atrial fibrillation (AF) and was reviewed by the acute medical team. Examination at this point revealed a congested, discoloured area of skin over the thenar eminence of her right hand. The combination of a cool hand and AF prompted an acute vascular referral.

On surgical review approximately two hours later, the patient was afebrile, hypotensive, (BP 100/70) and tachycardic, (pulse 120). The discolouration now covered all of the palmar aspect of her hand and extended to the midpoint of her forearm on the flexor surface. It was exquisitely tender to touch and palpation revealed crepitus. Good arterial signals were detected over radial and ulnar arteries with a hand-held Doppler device. A diagnosis of gas gangrene was made, and the plastic surgery team involved. Further examination demonstrated a football sized mass arising from the right pelvis, and local peritonism. An urgent erect chest x-ray showed free gas under her diaphragm.

The patient's transfer to theatre was expedited and the patient given broad-spectrum antibiotics. Surgical exploration demonstrated gangrenous muscles at the antecubital fossa. An above elbow amputation was performed. At laparotomy faecal peritonitis was found to be due to a perforated, but small caecal tumour, which was resected. An end ileostomy and mucus fistula were fashioned. The large pelvic mass appeared to be uterine fibroids.

The patient was transferred to Intensive Care sedated and ventilated and requiring inotropic support. Despite maximal therapy the patient deteriorated with multi-organ failure and succumbed two weeks later. There was however no further evidence of myonecrosis.

**Figure 1:**
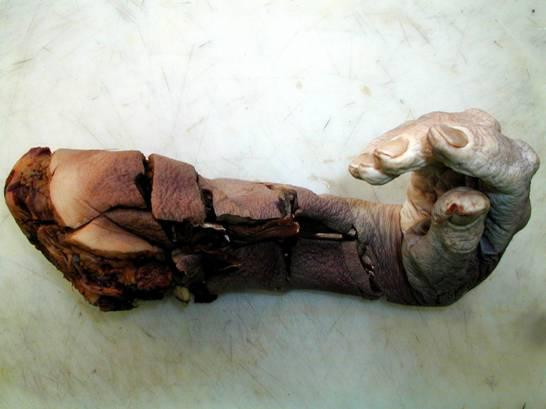
**Patients amputated right arm post dissection.** Some of the brown discolouration is visible.

Histology showed that the caecum contained an adenocarcinoma causing the perforation, and the forearm showed gas gangrene (figure 1). Intra operative cultures from both sites confirmed the presence of *Clostridium septicum*. Post-mortem studies revealed no further malignancy or evidence of gangrene.

## Discussion

The mechanism of metastatic myonecrosis is unclear. It is presumed *Clostridium septicum *gains access to the blood stream via a breach in the mucosal wall of the bowel. This process is aided by the presence of a lesion such as a malignancy or diverticular disease. The presence of immunosuppression in the form of diabetes or haematological abnormality aids the spread of infection.

Kornbluth et al reviewed many of the case reports in the literature in 1989 [2]. In total the findings of 162 cases of spontaneous *Clostridium *infection were presented, and 81% had an associated malignancy. In 34% of cases, this was a colon cancer. Distant, or metastatic, myonecrosis was reported as being the most lethal form, with 79% of patients dying. There were 38 patients with this condition, and of those 19, 50%, had a colonic malignancy.

Our review of available cases in the English literature since 1989 has found 28 cases, including our own, of *Clostridium septicum *myonecrosis [3-16]. Of these cases only 10 survived to discharge. 19 of the 28 (68%) were found to have a colorectal malignancy. 11 of the 28 patients were diabetic. There was no difference between type I and type II diabetes. The literature supported the relationship with haematological abnormalities, describing four patients with various malignant or immunosuppressive disorders [6,9,11,13].

We found 16 cases similar to our own, presenting with bowel pathology as a source of *Clostridium septicum *infection and metastatic myonecrosis [3,5,6,8,10,11,14-16]. Kornbluth et al reported a 79% mortality in this group prior to 1989. However, the literature we have reviewed since shows only 8 of 16 died, giving a mortality rate of 50%.

Although the gas gangrene described here is referred to as non-traumatic, there is some evidence from the literature that minor trauma could act as a focus for infection in susceptible individuals. Cases described include venous cannula sites, central venous catheter sites [8], and intramuscular injections [16]. It may well be that additional cases could be attributed to trivial trauma.

Given the relationship between colorectal cancer and myonecrosis, we would suggest extensive investigation in any patient who presents with *Clostridium septicum *myonecrosis, or has positive cultures. Our review suggests that right-sided cancers are more common, therefore the whole lower gastrointestinal tract should be visualised or imaged radiologically. Further to this, all patients should be screened for diabetes and haematological malignancies.

There was no convincing evidence for the use of hyperbaric oxygen therapy in the acute treatment of this form of gas gangrene, but some reports have extrapolated from its use in *Clostridium perfringens *infection and suggested that it forms an important part of the triad of antibiotics, surgical debridement and oxygen.

## Conclusion

In summary *Clostridium septicum *remains a destructive and rapidly lethal condition. The interesting aspect of this case is the unusual site of gas gangrene. Early surgical debridement and treatment with penicillin remains the key to success. Surviving cases should be screened for diabetes, haematological abnormalities and colorectal malignancies.

## Consent

Written informed consent could not be obtained in this case since the patient is deceased and there are no next of kin that we are aware of. We believe this case report contains a worthwhile clinical lesson which could not be as effectively made in any other way. We expect the next of kin would not object to the publication of this case. All efforts have been made to ensure that the risk of identification of this patient has been minimised to prevent the identity of the patient being revealed either to others or to the patient's relatives.

## Competing interests

The author(s) declare that they have no competing interests.

## Authors' contributions

MP wrote the case presentation and put together the paper. KS reviewed the previous literature. CM critically reviewed and edited the paper. All authors were involved in the care of the patient and all authors have reviewed the final document.

## References

[B1] Stokes E, Ridgway GL, Wren MWD (1993). Clinical Microbiology.

[B2] Kornbluth AA, Danzig JB, Bernstein LH (1989). *Clostridium septicum *infection and associated malignancy: A report of two cases and review of the literature. Medicine.

[B3] Ray D, SD Cohle, P Lamb (1992). Spontaneous *Clostridial *Myonecrosis. J Forensic Sci.

[B4] Burke MP, Opeskin K (1999). Nontraumatic *Clostridial *Myonecrosis. Am J Forensic Med Pathol.

[B5] San ildefonso A, Maeuri I, Facal C, Casal E (2002). Clostridium septicum infection associated with perforation of colon diverticulum. Rev Esp Enferm Dig.

[B6] Lorimer JW, Eidus LB (1994). Invasive *Clostridium septicum *Infection in Association With Colorectal Carcinoma. Can J Surg.

[B7] De Gara CJ, Mandel LA (1994). Invasive *Clostridium septicum *Infection in Association With Colorectal Carcinoma. Can J Surg.

[B8] O'Rourke J, Fahy C, Donnelly M (2003). Subcutaneous emphysema at the site of central line placement due to haematogenous spread of *Clostridium septicum*. Eur J Anaesthesiol.

[B9] Tehrani H, Gillespie PH, Cormack GC (2004). Fatal multifocal metastasis of *Clostridium septicum*: a case report. British Journal of Plastic Surgery.

[B10] Fernandez RJ, Gluck JL (1994). *Clostridium septicum *Gas Gangrene of the Gluteus Maximus and an Ascending Colon Malignant Tumour. Clin Orthop Relat Res.

[B11] Valentine EG (1997). Non-traumatic Gas Gangrene. Ann Emerg Med.

[B12] Salanitri GC, Tauro PG (1999). *Clostridium septicum *septicaemia with myonecrosis. Australas Radiol.

[B13] Agustin ET, Febre E, Brody GM, Liametz A, Cunha BA (1994). *Clostridium septicum *syndrome. Ann Emerg Med.

[B14] Stevens DL, Musher DM, Watson DA, Eddy H, Hamill RJ, Gyorkey F (1990). Spontaneous, Nontraumatic gangrene due to *Clostridium septicum*. Reviews of Infectious Diseases.

[B15] Sjolin SU, Hansen AK (1991). *Clostridium septicum *Gas Gangrene and an Intestinal Malignant Lesion. J Bone Joint Surg Am.

[B16] Kudsk KA (1992). Occult gastrointestinal malignancies producing metastatic Clostridium septicum infections in diabetic patients. Surgery.

